# Professional Tricksters

**Published:** 1861-07

**Authors:** 


### PROFESSIONAL TRICKSTERS.

Can it be possible that the honored and honorable diploma or license in physic should ever become a stalking-horse for trickery? Is it reasonable to suppose that the doctor would at any time be influenced by the petty sentiments of spite and envy, in his conduct toward his professional brethren? As a philosopher, or at least as a man of common sense, (a less pretentious, but by no means less creditable character,) it might be imagined that he was too intimately acquainted with the chances of life ever to think it worth while to feel anything but the deepest interest in their honor and welfare. Rightly appreciated, the success of another adds lustre to ourselves. The distinguished conduct of each conduces to the distinction of all, just as the prosperity of all contributes to the well-being of each. In this light there would be no place for envy, and the noblest of professions, standing among the first in science as it is the first in benevolence, disinterestedness, and fraternal kindness, would not be sullied by those bickerings and heartburnings which too commonly beset the course of every-day life. But, alas for human nature!

In yonder row of spacious mansions resides, no matter whence his wealth, a very rich man, who has purchased two of the houses and thrown them both into one. He is so rich that he consumes upon himself alone more than twice as much as any other ordinary human being, and, singular to say, enjoys better health than most mortals. His grooms, his horses, his furniture, and his household, all bespeak the man of money. He is a rare child of fortune, and fortune is good so long as she lasts. But what of that? He is sometimes ailing, and in the hour of need, real or imaginary, resorts to the aid of medical skill. What a pluming of pinions among the rising M.D.'s! What a shuffling of feathers among the eager general practitioners! What tiptoe excitement to learn upon whom will fall the patronage of one who is as much beneath them in intelligence as he is above them in wealth! Of course, only one can be selected. The rest bite their lips and retire. The great man takes a dislike to the one he has chosen. He turns him off and calls in a second, who is in turn dismissed as summarily as the first. Both the first and the second were men of approved talents and probity; but the great man does not care for that; they did not suit him, and he puts them aside at a moment's notice. He at last falls in with one to his entire satisfaction, in the person of an ignoramus as clever as himself. It is a decided hit; they were made for each other; and Dives and Ignoramus go hand in hand. The squad of the rejected look on and wonder, but it is a wonder to no one except themselves.

In that well-furnished nursery lies a sick child, tended by its officious nurse, and watched by its sensitive mamma with continued and restless solicitude. The care bestowed upon the infant is out of all proportion to the exigency of the case. The child is ill and may possibly die, but will, under ordinary care and attention, in all probability recover. The medical man who has charge of the case is a well-informed and experienced practitioner, perfectly aware of the contingencies of the ailment, and calmly alive to the whims and fancies by which he is beset. His little patient lingers on; his credit is on the wane. Another practitioner is named of infallible skill, particularly in cases of this description; and he is called into consultation along with the family medical attendant. At the appointed hour, a carriage and pair drive up to the house; no knocker is raised, for fear of a noise; only the door-bell vibrates gently; and in walks the pattern M.D. He is a tall man with an obsequious stoop, and his knees slightly bent. His hair is brushed back; he wears gold spectacles, a white tie, and a black suit. There is no creaking of his shoes, and his manner is bland and soothing. He hangs over the crib of the dear sick child in a solemn attitude of observation; touches it lightly, listens to its breathing, feels its tiny pulse at the wrist, and then, quietly looking up, asks the old practitioner, who is standing by and looking on, whether he has given his little patient *Tous les mois*—a panacea at that time only just introduced. The answer is in the negative. What?—Not!—replies the pattern, with an affected look of surprise; not given *Tous les mois?* *Tous les mois*, nurse; *Tous les mois*, my lady—turning to the agonized mamma—*Tous les mois* will cure your child! The old practitioner is dismissed, on the score of ignorance, and under the judicious use of *Tous les mois* the child recovers.

There are tricks in every trade, but of all tricks, professional pedantry is the most detestable. It has it all its own way. The party duped can have no insight into the secrets by which he is guided in the management of his property, his soul, or his life. He must trust implicitly to the integrity and skill of his professional adviser, whom he flies to in moments of the last resort. It is in the embarrassment of such occasions that the trickster succeeds. There is the opportunity of putting himself forward, and he seizes it with adroit avidity.

The *monstror digito prœtereuntium* is a vanity common to all sorts of professional persons—the painter, the musician, the poet, the medical man, and artists of every description. It is gratifying to have ourselves pointed out as we pass along, and hear it said, that is he: *pulchrum est digito monstrari et dicier Hic est*, as Tacitus says, speaking of the orators. It is a venerable folly, sustained by classical authority, countenanced by Cicero, and quizzed by Horace. A slave nudged the aspirant for public favor in the side, (*fodiat latus*,) and whispered in his ear the name and title of those who met or passed him in the way. It is the same now as it was then. The rising medical man does not indeed retain a nomenclator at his elbow, but he does what is equally as effective, for he publishes a book to make himself known, and he rides in a carriage to let himself be seen. The world will not step out of its way to look after you. If you are not known, you may as well be lost or dead. Unadvertised, you go for nothing. You must proclaim your own merits, and put forward your own pretensions, with the best grace you may, or else make up your mind to subsist on modesty, truth, and small means for the rest of your days.

The competition which such a line of conduct necessarily provokes engenders the heartburnings and petty rivalries that ruffle the social surface of the medical world. It is the old story—there cannot be two Cæsars in the same camp, nor two Kings of Brentford in the same village—*omnis potestas impatiens consortis*, and in the medical profession this is particularly the case. There can be no partnership in medicine, for it turns upon personal merit; if one succeeds the other fails, and the one that fails envies or hates his successful rival. Consequently, unless the mind and temper be extremely well regulated, the egotism and self-sufficiency of the medical character are almost proverbial. Who ever supposes that doctors were framed for the love of one another? No one, unless he be a simpleton or a novice. Men of the world suppose no such thing. They take it for granted that two of a trade never agree. Hence it is that, though unfounded in fact or reason, medicine holds so low a place in their esteem. Without giving themselves the trouble of examining the essential difference between charlatanism and science, they *lump* the whole together, and regard it all as a mystery or pretense, which they must use as well as they can, whenever the hour for having recourse to it is forced upon them. Were it not for this adverse impression, medicine would long ago have gained the ear and secured the attention of government, upon the ground of its being a practical science of the highest national importance.

People are misled by appearances. That general practitioner you see driving along in his open barouche was educated at a small preparatory school, served his time in an apothecary's shop, passed his examinations, and is now playing the fine gentleman. He never attends a servant, nor any inferior person—*i.e.* an unpaying patient, except as an act of grace. No one could be more timid than he was on his first commencing practice. A child might have whipped him with a straw. At this critical juncture he besought the aid of one much older, if not wiser, than himself, and in him found a friend who fulfilled indeed the adage of being a friend in need. Night and day he beset his friend's door, which was always open to his call, and there he found, what he required, help in his necessities, consolation for his alarms, and the assurance of his final success. The hour of success arrived as it had been predicted; but no sooner had it arrived than he kicked the ladder that had helped him up from under his feet, mounted the platform, as it were, by his own unaided efforts, and stood alone. Now he lords it among the best of his fellows. Only a few are good enough to be countenanced as his equals. His manners and deportment are supercilious and overbearing. He takes the upper hand with his brethren, except those to whom he thinks proper to bow, to cringe, or to court. Colbert, in the reign of Louis XIV., did the same, and so did Sir Andrew McSycophant, in the *Man of the World.* It is by no means a bad game, although it sometimes fails egregiously. As to his absolute knowledge and acquirements, they are as shallow as shallow can be, but perfectly suitable to that class of delicate cases in which the married and unmarried in brocade and finery are supposed to rule supreme. His behavior is what is emphatically styled *sugary,* and he is a lady's man in every sense of the word.

The puppets that strut upon the stage of the world are as numerous and amusing in medicine as they are in any other profession, trade, or calling. Perhaps more so, because the practice of physic ministers directly to the self-love and egotism of its votaries. None but his admirers and friends seek the popular practitioner; he never knows his enemies, who never consult him. He moves in a charmed circle, within the sphere of which he meets with nothing but adulation, and beyond which he never steps. He is a pet in the fullest meaning of the word. His little world cannot live without him, till it has changed its mind, and then he must learn to live without it.

The stage upon which he struts has its loose boards, which sometimes slip from beneath his feet before he is aware of it. It is his ignorance of the jeopardy in which he is placed that makes him forget himself and pretend to be something great. He cannot see, nor does he know, that so long as his career lasts, he is nothing better than an actor dressed up for the hour to suit the nonce. He calls down the plaudits of the house whenever he appears on the boards. But let him quit the scene, and walk the streets by daylight as a private man, and he will find that no one knows him in his plain clothes. The applause which made him giddy was due to his office, not to himself. The next that treads in his steps will succeed as ably as he has done, and fail or fall as quickly as himself. Professional friendship is the expression of public confidence. As such it is invaluable, since it is, in fact, only another term for character and reputation; but overrate it, and you will find to your cost that, like the ass in the fable, you have made a false step by leaping into your master's lap.

There is a ripe season in every one's life, which soon comes to a close. The summer solstice is brief. The best man has his day. The best actor may linger too long upon the stage. The *vivas* of yesterday will be re-echoed by the *à bas* of to-morrow.

The same shield has its two sides, the golden and the leaden one. We have been looking at the lead; now let us see the gold. In an age which was none of the brightest Charles II. did not hesitate to reward the author of the *Religio Medici*, and the good and quaint Sir Thomas Browne still adorns the list and library of the Royal College of Physicians. The writings of Sydenham are the best comments on himself, and his worth has obtained for him the inestimable title of the Father of English Medicine. The evening of life closed in upon Boerhaave, and found him in the vale of years still studious and religious, endeavoring to dispense for the maladies of others that relief which he had failed to procure for himself. It is superfluous to expatiate on the virtues of these great men. They are known to all the world. But if there were giants in those days, the men of modern date are not pigmies. The names of Babington, Bailey, Cooper, and Abernethy are as household words upon our lips; and their successors, Brodie, Bright, Watson, Copland, Todd, and others, whom it would be as invidious to omit as to mention, deserve no less a meed of praise. Their career is graceful and attractive, because it is their own. They are what they are, and no change of fortune could dwarf their just proportions. Medicine owes them everything. Good sense and good taste, sound learning and practical skill, modesty and worth, are qualities which compel universal respect, defy criticism, and outlast time.

Dignum laude virum Musa vetat mori.

(*Medical Critic and Psychological Journal*, April, 1861.)

